# HOXB5 Cooperates with NKX2-1 in the Transcription of Human *RET*

**DOI:** 10.1371/journal.pone.0020815

**Published:** 2011-06-03

**Authors:** Jiang Zhu, Maria-Mercedes Garcia-Barcelo, Paul Kwong Hang Tam, Vincent Chi Hang Lui

**Affiliations:** 1 Department of Surgery, Development & Growth, LKS Faculty of Medicine, The University of Hong Kong, Hong Kong SAR, China; 2 Centre for Reproduction, Development & Growth, LKS Faculty of Medicine, The University of Hong Kong, Hong Kong SAR, China; University of South Florida, United States of America

## Abstract

The enteric nervous system (ENS) regulates peristaltic movement of the gut, and abnormal ENS causes Hirschsprung's disease (HSCR) in newborns. HSCR is a congenital complex genetic disorder characterised by a lack of enteric ganglia along a variable length of the intestine. The receptor tyrosine kinase gene (*RET*) is the major HSCR gene and its expression is crucial for ENS development. We have previously reported that (i) HOXB5 transcription factor mediates *RET* expression, and (ii) mouse with defective HOXB5 activity develop HSCR phenotype. In this study, we (i) elucidate the underlying mechanisms that HOXB5 mediate *RET* expression, and (ii) examine the interactions between HOXB5 and other transcription factors implicated in *RET* expression. We show that human HOXB5 binds to the promoter region 5′ upstream of the binding site of NKX2-1 and regulates *RET* expression. HOXB5 and NKX2-1 form a protein complex and mediate *RET* expression in a synergistic manner. HSCR associated SNPs at the NKX2-1 binding site (-5G>A rs10900296; -1A>C rs10900297), which reduce NKX2-1 binding, abolish the synergistic trans-activation of *RET* by HOXB5 and NKX2-1. In contrast to the synergistic activation of *RET* with NKX2-1, HOXB5 cooperates in an additive manner with SOX10, PAX3 and PHOX2B in trans-activation of *RET* promoter. Taken together, our data suggests that HOXB5 in coordination with other transcription factors mediates *RET* expression. Therefore, defects in *cis-* or *trans-*regulation of *RET* by HOXB5 could lead to reduction of *RET* expression and contribute to the manifestation of the HSCR phenotype.

## Introduction

In mammalian embryos, neural crest cells (NCC) migrate from the neural tube, enter the foregut and colonize the gut, where they differentiate into neurons and glia of the enteric nervous system [1]. The enteric nervous system (ENS) comprises a network of neuronal ganglia and glia within the gut wall, which controls gut peristalsis. In human, abnormal ENS development results in absence of ganglia at the caudal-most gut in newborns with Hirschsprung disease (HSCR), and patients develop a life-threatening condition of intestinal obstruction due to defective peristalsis. HSCR is a complex oligogenic disease and a major ENS developmental disorder affecting newborns with variable incidence in different races, and is most often found in Asians (28 per 100,000 live births) [2].

The receptor tyrosine kinase gene RET encodes a receptor for glial cell-line derived neurotrophic factor (GDNF) on NCC and is crucial for ENS development [3]–[6]. RET is the major HSCR gene, and loss-of-function mutations in RET account for up to 50% of familial and 7–35% of sporadic cases of HSCR [7]–[12]. Other genes implicated in HSCR that account for 7% of cases encode proteins involved in signaling pathways such as the endothelin 3/endothelin receptor B, and transcription factors SOX10, PHOX2B, NKX2-1 (also known as TITF-1), which govern ENS development [1], [13], [14]. Some of these transcription factors, for example NKX2-1 and SOX10 bind to cis-regulatory elements of the RET gene and regulate RET expression [13], [15], [16]. Single nucleotide polymorphisms (SNPs) affecting the binding and regulatory activities of NKX2-1 and SOX10 on RET were found associated with a reduced expression level of RET in patients and increasing risk of HSCR development [17], [18]. These data indicated that the manifestation of the HSCR phenotype may result from the interaction between altered genes of these signalling pathways, which may ultimately result in an altered expression of RET, and consequently in the HSCR phenotype.

In human and mouse embryonic guts, Homeobox (*HOX*) gene *HOXB5* is expressed in both migrating NCC and the gut mesenchyme, and the expression pattern of *HOXB5* is associated with the migration and differentiation of NCC, suggesting a unique role of *HOXB5* in ENS and gut musculature development [19]–[22]. To investigate *Hoxb5* function in ENS development and to circumvent the problem of functional redundancy, we generated transgenic mice that can be induced by *Cre* to express a chimeric protein (enb5) in NCC. In this, the DNA-binding homeodomain of Hoxb5 is linked to the repressor domain of the *Drosophila* engrailed protein. This chimeric enb5 repressor competes with wild-type Hoxb5 for binding to target genes and inhibits transcription, thereby blocking the developmental pathways that normally require Hoxb5. We showed that the Hoxb5 trans-activated *Ret* expression, and that perturbation of Hoxb5 function caused down-regulation of Ret in NCC resulting in defective NCC migration in the developing gut and HSCR phenotype in *enb5/Cre* transgenic mice [23]. Our data indicate *Ret* is a downstream target of Hoxb5 whose perturbation causes reduced Ret expression, impairs NCC migration and leads to ENS phenotypes. In addition, our case-control study suggests *HOXB* locus may affect the penetrance of the HSCR [24]. Therefore, it is crucial to delineate the underlying mechanisms HOXB5 regulate *RET* expression to unravel the roles of HOXB5 in the etiology of HSCR.

In this study, we show that human HOXB5 physically binds with the promoter of the human *RET* gene and regulates *RET* expression. HOXB5 and NKX2-1 form protein complex and work synergistically in the activation of *RET* expression. The synergistic interaction of HOXB5 and NKX2-1 in the activation of *RET* expression is compromised by promoter SNPs, that disrupt the NKX2-1 binding to the *RET* promoter.

## Results

### HOXB5 trans-activates RET promoter

**Figure 1:**
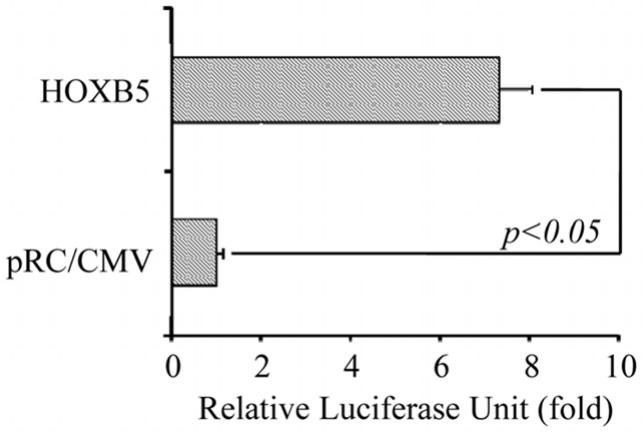
HOXB5 trans-activates human *RET* gene. Trans-activation activity of HOXB5 on *RET* promoter was assayed by measuring the luciferase activity from human *RET* promoter luciferase reporter construct. Luciferase activity was normalized with Renilla luciferase to obtain relative luciferase unit. Data are shown as the fold increase (mean±SD) in relative luciferase unit compared to that of pRC/CMV.

**Figure 2:**
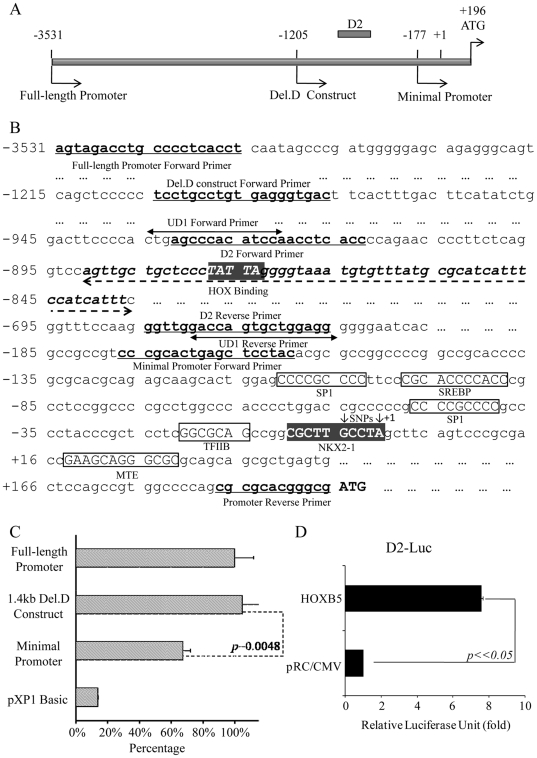
Localization of cis-regulatory element in *RET* promoter responsible for HOXB5 trans-activation. A, Schematic diagram of the human RET promoter. “+1” denoted transcription start of *RET* gene, and the first ATG of *RET* gene was indicated. The 5′ ends of sequential deletion constructs of *RET* promoter were indicated with nucleotide positions. Location of the D2 element was indicated. B, Nucleotide sequence of the human *RET* gene promoter was shown with the locations of various primers used for the generation of deletion constructs. Primers pair used for the generation of D2 PCR fragment for EMSA, and for the PCR amplification in ChIP assays were indicated. The PCR primers (UD1 forward and reverse primer) used for real-time quantitative PCR for the binding of D2 to HOXB5 were indicated with ↔. The D2A oligonucleotide probe (bold and italic) which was specifically bound by HOXB5 as shown by EMSA was underlined with dotted arrow, and the putative HOXB5 binding site in D2 was indicated by filled boxed. NKX2-1 binding site was boxed, and the two SNPs affecting NKX2-1 binding were indicated with downwards arrows. Putative binding sites of other transcription factors were boxed and labeled accordingly. Abbreviations: TFIIB, transcription factor II B; SREBP, sterol regulatory element-binding protein; MTE, human motif ten element. C, Trans-activation of HOXB5 on full-length and 5′ deletion *RET* promoter was assayed by measuring the luciferase activity from *RET* promoter luciferase reporter constructs. Luciferase activity was normalized with Renilla luciferase to obtain relative luciferase unit. Percentage trans-activation of deletion constructs was determined by comparing luciferase activity to that of full-length promoter construct, which was arbitrarily regarded as 100%. D, Trans-activation of HOXB5 from D2 was assayed by luciferase activity from a D2-SV40 promoter luciferase reporter. Luciferase activity was normalized with Renilla luciferase to obtain relative luciferase unit. Fold increase (mean±SD) was determined relative to luciferase unit of pRC/CMV which was arbitrarily regarded as 1.

Our group has previously reported that Hoxb5 induces transcription of *Ret* in mice [23]. To study if HOXB5 protein trans-activates human *RET* promoter, we examined the transcriptional activity of HOXB5 on a luciferase reporter construct consisting of 3.7kb human *RET* promoter sequence 5′ upstream of luciferase gene. SK-N-SH neuroblastoma cell was shown to express *RET*, *NKX2-1*, *PHOX2B*, but not *SOX10*, *PAX3* [25]. In addition, SK-N-SH cell also did not express *HOXB5* (our unpublished data). Therefore, SK-N-SH cell was chosen to examine the regulatory role of HOXB5 on *RET* gene. HOXB5 displayed a 7-fold induction of transcription from human *RET* promoter, as compared to empty vector control pRC/CMV (Figure 1). *In silico* analysis identified HOX consensus binding motif in the promoter of human *RET* gene, suggesting that HOX may regulate *RET* expression by binding to the promoter (Figure 2A, B). To delineate the cis-element in the promoter responsible for HOXB5 induction, 5′ deletion reporter constructs spanning −1205 to +196 (Del.D), and −177 to +196 (minimal promoter construct) were generated and tested for HOXB5 induction. HOXB5 induced luciferase activity from Del.D comparable to that from the full-length promoter. In contrast, luciferase activity from the minimal promoter construct was significantly lower than those from the full-length and Del.D reporter constructs (Figure 2C), which suggested that the region spanning from −1205 and −177 was important for HOXB5 induction.

**Figure 3:**
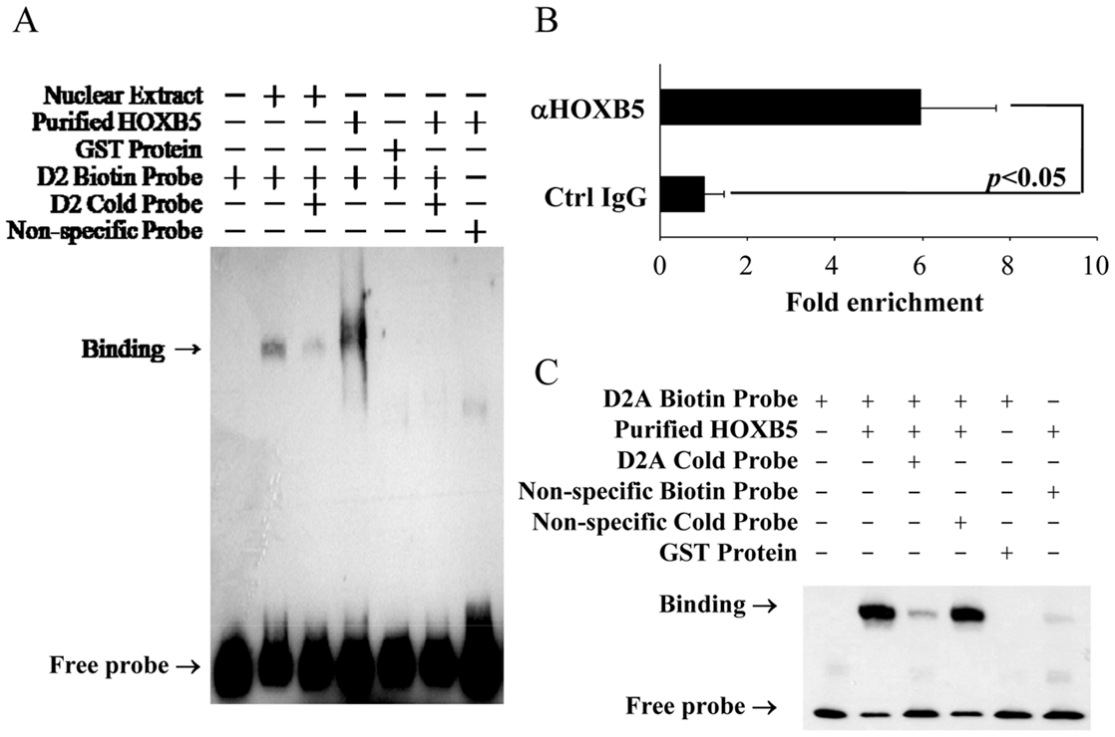
HOXB5 physically associates with human *RET* promoter. A, EMSA assay with D2 and nuclear extract of HOXB5 transfected SK-N-SH cells or purified GST-HOXB5 protein. Binding of D2 as indicated by retarded migration of D2 biotin probe (arrow) was specifically detected in lanes with *HOXB5* transfected SK-N-SH nuclear extract or purified HOXB5 protein. Addition of excess unlabeled D2 probe (D2 cold probe) either reduced or completely abolished the binding of labeled D2 probe. B, ChIP was performed with HOXB5 transfected SK-N-SH cells, and binding of D2 to HOXB5 was determined by real-time quantitative PCR using UD1 forward and UD1 reverse primer. x-axis indicated fold enrichment normalized to the control IgG. C, Binding of D2A oligonucleotide to purified HOXB5 protein as indicated by retarded migration of D2A probe. Addition of excess unlabeled D2A probe reduced the binding markedly. No competition was observed with the addition of non-specific unlabled probe, and no binding was detected with GST protein.

Overlapping PCR fragments spanning the promoter region (−1205 to −177) were tested for HOXB5 trans-activation to demarcate the HOXB5-responsive element. It was found that HOXB5 induced luciferase activity from D2 fragment (spanning −932 to −666) with heterologous SV40 promoter (Figure 2D; D2-Luc). To directly confirm that HOXB5 enhances luciferase expression by physical association with D2, EMSA and ChIP analysis were performed in *HOXB5* transfected SK-N-SH cells. D2 fragment was shown to bind to HOXB5 as revealed by EMSA. A Protein-DNA complex was observed when nuclear extract or HOXB5-GST fusion protein was incubated with biotin labeled D2 fragment probe (D2 probe), and this binding was abolished when 100-fold molar excess of cold probe was added (Figure 3A). ChIP analysis was performed with an antibody to HOXB5; binding to D2 was detected by real-time quantitative PCR. Our result showed that binding of HOXB5 to D2 was enriched compared to the non-specific IgG control (Figure 3B). Furthermore, EMSA showed that HOXB5 bind to the oligonucleotide D2A that contain the HOX binding sequence (Figure 3C). HOXB5 was found neither associate with nor trans-activate from the other PCR fragments (Figure S1).

### HOXB5 synergizes NKX2-1 in RET transcription

**Figure 4:**
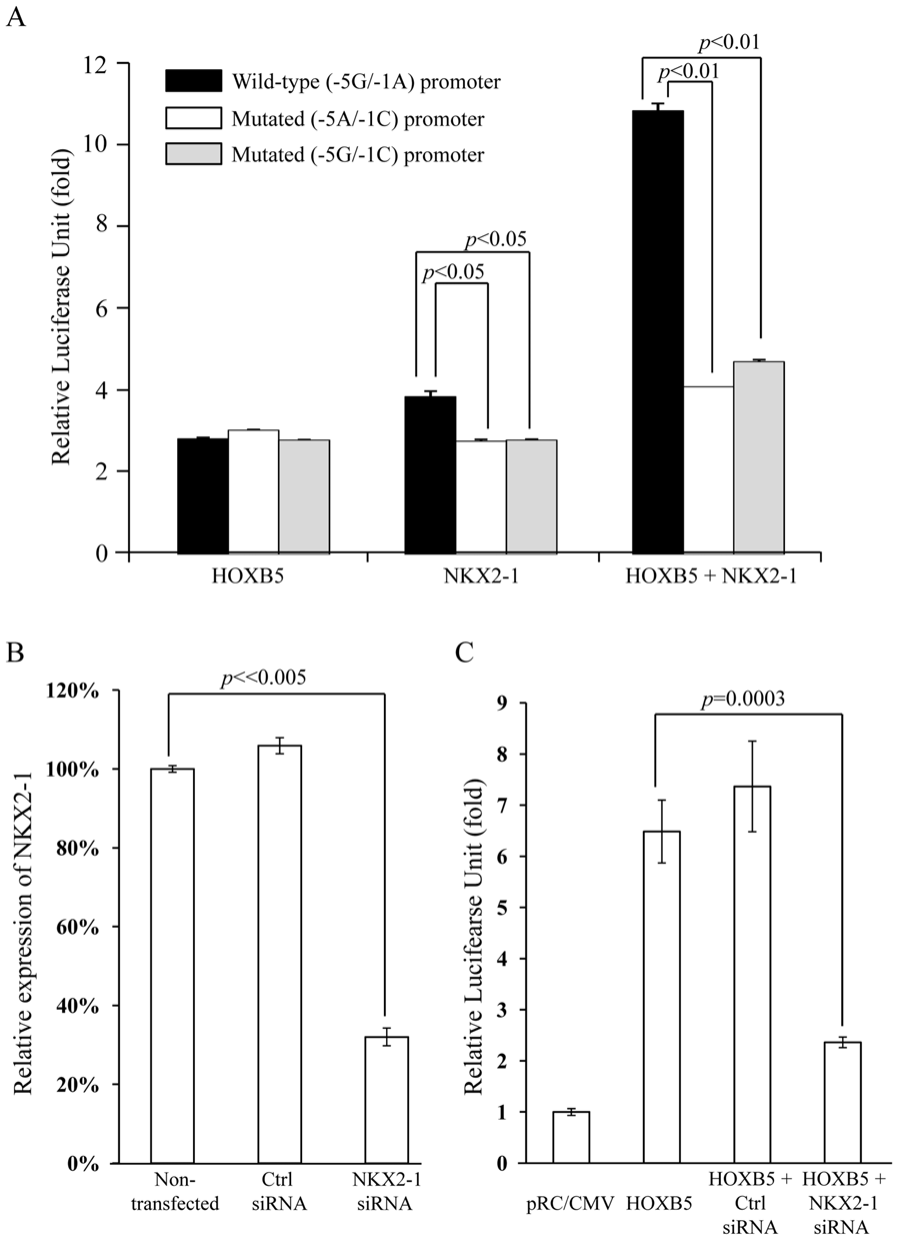
HOXB5 synergizes NKX2-1 in trans-activation of human *RET* gene. A, Wild-type or mutated (SNPs -5G>A and -1A>C) or (-1A>C) full-length *RET* promoter was transfected with HOXB5 or NKX2-1 alone or in combination into SK-N-SH cells. Relative luciferase units for each combinations were determined, and fold increase (mean±SD) was calculated as compared to luciferase unit of empty vector (pXP1 Basic), which was arbitrarily regarded as 1. B, Expression levels of *NKX2-1* mRNA were determined by real-time RT-PCR quantitation in non-transfected SH-N-SH cell and SH-N-SH cell transfected with *NKX2-*1 siRNA or negative control siRNA (Ctrl siRNA). Relative expressions of *NKX2-1* were calculated with reference to non-transfected cell, which was arbitrarily regarded as 100%. C, full-length *RET* promoter was transfected with HOXB5 alone or in combination with *NKX2-1* siRNA or negative control siRNA (Ctrl siRNA). Relative luciferase units for each combination were determined, and fold increase (mean±SD) was calculated as compared to luciferase unit of empty vector (pRC/CMV), which was arbitrarily regarded as 1.

Our group previously reported that NKX2-1 trans-activates the *RET* promoter and is important for the expression of *RET* [13]. HOX binding site is located 874 nucleotides upstream of NKX2-1 binding site in the *RET* promoter, suggesting that these transcription factors may cooperate in the regulation of *RET* transcription. *HOXB5* was co-transfected with *NKX2-1* into SK-N-SH cells to examine the cooperation between these two transcription factors in the trans-activation of *RET* promoter. The inductions were calculated as fold activations relative to the empty vector pRc/CMV. HOXB5 showed a 7.3-fold induction, and NKX2-1 displayed a 4.5-fold induction from the *RET* promoter. In contrast, 14.5-fold induction was observed when *HOXB5* and *NKX2-1* were co-transfected, which was significantly higher (p<0.05) than the sum of trans-activation of HOXB5 and NKX2-1 alone (Figure 4A).

Binding of NKX2-1 to *RET* promoter is sensitive to the promoter SNPs, which overlap the NKX2-1 binding site [13]. To examine if the promoter SNPs affects the synergistic cooperation between HOXB5 and NKX2-1, mutated full-length reporter constructs with the two SNPs (-5G>A and -1A>C) or one SNP (-1A>C) were examined for trans-activation by HOXB5 and NKX2-1. In line with our previous findings, SNPs (-5G>A and -1A>C) and SNP (-1A>C) reduced the trans-activation of *RET* promoter by NKX2-1 (Figure 4A). However, these SNPs did not affect the HOXB5 trans-activation. The synergistic cooperation of HOXB5 and NKX2-1 in the trans-activation from the *RET* promoter was reduced drastically by the SNPs. We further knockdown the endogenous NKX2-1 expression in SK-N-SH by siRNA. Reduction of NKX2-1 expression by 70±2% (mean±SD; *p*≪0.005; n = 3) (Figure 4B) resulted in a significant reduction of HOXB5 trans-activation of *RET* promoter by 68.7±7% (mean±SD; *p* = 0.0003; n = 3) (Figure 4C). Synergistic interaction was not observed between HOXB5 and other transcription factors PHOX2B, SOX10 and PAX3, that have been shown capable of trans-activating *RET* promoter. Instead, only additive effects were observed between HOXB5 and SOX10 in the trans-activation of *RET* promoter (Figure S2).

**Figure 5:**
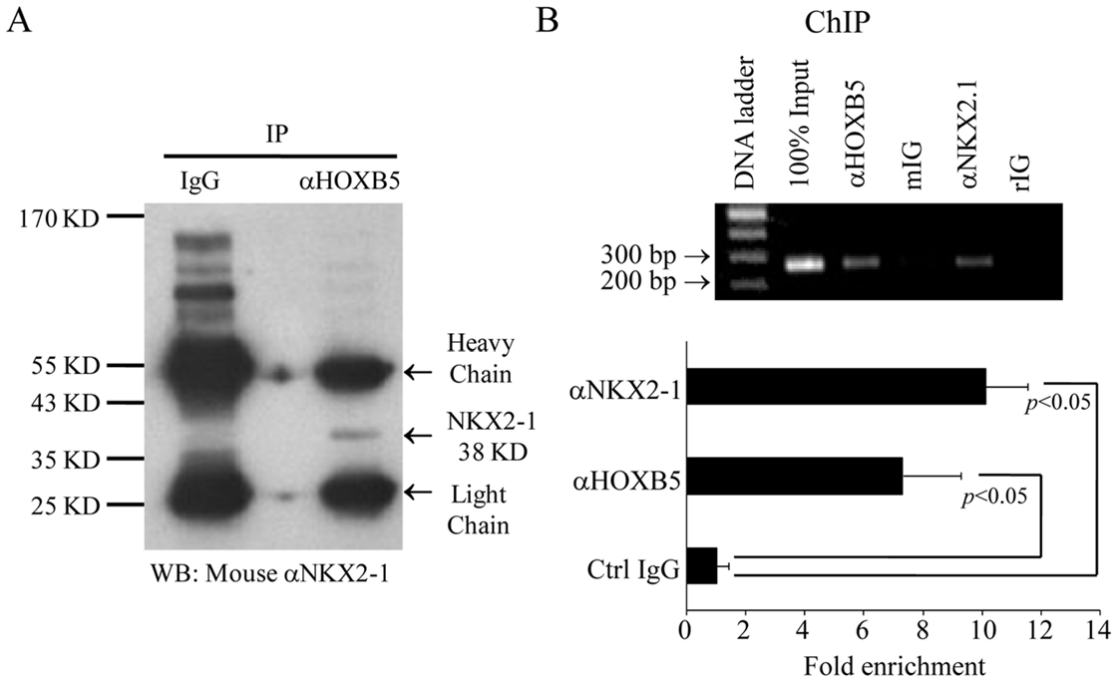
HOXB5 and NKX2-1 form a protein complex binding to *RET* promoter. A, Immuno-precipitation (IP) on nuclear extract of HOXB5 transfected SK-N-SH cells with αHOXB5 antibody. Protein complexes precipitated with αHOXB5 or non-specific IgG were resolved by SDS-PAGE, and NKX2-1 in the complex was detected by immuno-blotting using αNKX2-1 antibody. NKX2-1 (38KD) was specifically detected in the lane of protein complex precipitated with αHOXB5. B, Chromatin immuno-precipitation (ChIP) of *HOXB5* transfected SK-N-SH cells and αHOXB5 or αNKX2-1 antibody. D2 was amplified from chromatin precipitated with αHOXB5, and αNKX2-1, but not in the preparation with non-specific IgGs (mIG; rIG). Bindings of HOXB5 and NKX2-1 to D2 were detected by real-time quantitative PCR. *x* axis is fold enrichment normalized to the non-immune mouse IgG control.

The observation that HOXB5 and NKX2-1 bound to the *RET* promoter, and HOXB5 synergized NKX2-1 in the *RET* transcription, prompted us to investigate if HOXB5 physically associates with NKX2-1. Using antiserum against HOXB5 for pull-down and anti-NKX2-1 for the detection of NKX2-1 protein in the protein complex, NKX2-1 was co-precipitated with HOXB5 in the Co-IP experiment (Figure 5A). As revealed by ChIP analysis, the chromatin region containing D2 was precipitated with anti-HOXB5 and anti-NKX2.1 (Figure 5B). In addition, binding of D2 to HOXB5 and NKX2-1 was further confirmed by ChIP and quantitative PCR (Figure 5B). Taken together all these suggested that HOXB5 and NKX2-1 formed a protein complex and bound to the *RET* promoter.

## Discussion

Expression and proper function of RET are crucial for the development of ENS [3]–[6]. Loss-of-function mutations in *RET* have been identified in both familial and sporadic cases of HSCR [7], [8], [10]. Genetic variants in the *cis*-regulatory elements of *RET* also play a role in mediating individual susceptibility to HSCR, by compromising the *RET* expression [17], [18]. Therefore, to unravel the molecular mechanisms of the regulation of *RET* expression is critical in understanding the molecular etiology of HSCR.

The minimal promoter (−177 to +196) of the human *RET* gene contains regulatory elements for the binding of basal transcription factors essential for basal *RET* expression [26]–[28]. On the other hand, tissue-specific expression of *RET* is regulated by tissue-specific transcription factors, working cooperatively via interactions with their respective *cis*-regulatory elements in the *RET* promoter [15], [16], [25].

HOXB5 binds to the HOX consensus sequence at 875bp 5′ upstream of transcription start of *RET* gene, and deletion of the binding sequence abolishes HOXB5 trans-activation. HOXB5 and NKX2-1 form protein complex binding to the *RET* promoter, and work in a synergistic manner in mediating *RET* expression. Down-regulation of NKX2-1 by siRNA suppresses HOXB5 trans-activation of *RET* promoter, indicating that association with NKX2-1 is essential for HOXB5 in the regulation of *RET* expression. NKX2-1 binds at the region overlapping with the transcription start site, and mediates *RET* transcription [13]. HSCR associated SNPs at the NKX2-1 binding site (-5G>A rs10900296; -1A>C rs10900297) reduce NKX2-1 binding and trans-activation of *RET* [13], [14], [18], [29]–[31]. These promoter SNPs have no direct effect on HOXB5 trans-activation activity, but abolish the synergistic interaction between HOXB5 and NKX2-1 on *RET* transcription, indicating that binding of NKX2-1 to the promoter is required for HOXB5 and NKX2-1 to work synergistically in mediating *RET* expression. NKX2-1 is a developmentally regulated homeodomain transcription factor [32], and possesses two transcriptional activation domains that interacts with other proteins to regulate target gene transcription [33]. We have previously indentified a Gly→Ser mutation in the NKX2-1 transcriptional activation domain in one HSCR patient. This mutation (Gly322Ser) reduces the NKX2-1 induction of *RET* promoter without affecting its binding onto the *RET* promoter, suggesting that the Gly→Ser mutation may interfere with the protein-protein interactions of NKX2-1 with other transcription factors [14]. Further experiments are needed to ascertain if HOXB5 interacts with NKX2-1 via these transcriptional activator domains.

Our data show only an additive effect between HOXB5 and SOX10 on the trans-activation from the full-length *RET* promoter containing the SOX10/PAX3-responsive element. In line with a lack of synergistic interactions between HOXB5 with SOX10 and PAX3, HOXB5 exhibits comparable trans-activation from the full-length promoter and truncated promoter (Del.D construct) in which the SOX10/PAX3-responsive element is deleted. These data indicate that HOXB5 mediates transcription of *RET* independent of SOX10 or PAX3.

Although NCC express *SOX10*, *PAX3*, *HOXB5*, *NKX2-1* and *RET*, co-expression of these transcription factors and *RET* is not always found in all *SOX10*-, *PAX3*- or *HOXB5*-expressing tissues [15], [19], [34]. Furthermore, SK-N-SH cells express *RET* and *NKX2-1*, but not *SOX10*, *PAX3* and *HOXB5* [25] (Figure S3). Using dual luciferase-reporter assays, we have previously shown that NKX2-1 indeed works coordinately with PHOX2B and SOX10, but not PAX3, to mediate *RET* transcription [25]. It would appear that the expression of *RET* is regulated by a number of transcription factors including HOXB5, NKX2-1, SOX10, PAX3 and PHOX2B, and that none of these transcription factors is necessary or sufficient for *RET* transcription. Instead, these transcription factors work either in a synergistic and/or additive manner to mediate *RET* expression.

*RET* is repressed in most tissues and cells, and its expression is associated with a transition from a repressive chromatin to an open, permissive chromatin [35], [36]. It is plausible to speculate that association of HOXB5 with HOX binding sequence and interaction with NKX2-1 at the transcription start site, may induce conformational change of the *RET* promoter bringing transcription factors in close proximity of minimal promoter facilitating the recruitment of basal transcription factors mediating *RET* transcription.

In conclusion, we showed that i) HOXB5 binds to crucial *RET* regulatory region and affects *RET* expression; ii) HOXB5 synergizes with NKX2-1 transcription factor, and iii) HOXB5 regulatory activity is compromised by sequence variants within *RET* regulatory regions. Taken altogether, our data suggests that HOXB5,in coordination with NKX2-1, plays an essential role in *RET* expression. Future studies shall focus in delineating the interplay of molecular components implicated in the regulation of *RET* expression, which will provide important mechanistic insights into the pathogenesis of HSCR.

## Materials and Methods

### Cell culture

Human neuroblastoma cell line SK-N-SH (#HTB-11) (ATCC, Manassas, USA) was cultured in Eagle's minimal essential medium (EMEM) (ATCC, Manassas, USA) supplemented with 10% fetal bovine serum (FBS) (Gibco, Invitrogen, Carlsbad, USA) without antibiotics at 37°C and 5% CO_2_. Trypsin-EDTA solution (0.25%; w/v) was used for trypsinization of cells. Cells were stored in 5% (v/v) DMSO in liquid nitrogen.

### Luciferase-reporter constructs

The 3.7 kb *RET* full-length promoter (1545 to 5270, AF032124, Genbank), the 372 bp minimal promoter and the mutated *RET* full-length promoter containing -5G>A and -1A>C or -1A>C alleles, were generated by PCR and cloned upstream of the luciferase reporter in the pXP1 vector (AF093683, GenBank) [13]. All of these constructs contain a 195 bp 5′UTR of *RET* gene. A 1.4 kb 5′ deletion construct (Del.D), was generated by cloning of the PCR fragment (-1205 to 195) of *RET* gene upstream of the luciferase reporter in the pXP1 vector. Furthermore, D2A-Luc construct was generated by cloning of the PCR fragment (-932 to -666) of *RET* gene upstream of the luciferase reporter in the pGL3 vector (Promega, Madison, USA).

### Transient transfection and dual-luciferase reporter assay

SK-N-SH cells were seeded on 24-well 18 hours prior to transfection. Cells were co-transfected with pRL internal control, reporter constructs and appropriate amount *HOXB5* and/or *NKX2-1* expression vectors with Lipofectamine 2000 (Invitrogen, Carlsbad, USA). Cell lysate was analyzed 24 hours by Dual-Luciferase Reporter Assay System (Promega, Madison, USA) on MicroLumatPlus LB 96V instrument. At least two independent triplicate or quintupled experiments were performed, and the luciferase activity was presented as relative luciferase unit (RLU) normalized with the *Renilla* luciferase internal control.

### Small interference RNA knockdown of NKX2-1

Transfection of small interfering RNA (siRNA) was performed using Lipofectamine 2000 reagent (Invitrogen, Carlsbad, USA) according to the siRNA-plasmid co-transfection protocol from Invitrogen. Human *NKX2-1* siRNA (Stealth RNAi HSS144278) and negative control (Stealth RNAi 12935-200) were purchased from Invitrogen (Invitrogen, Carlsbad, USA).

### Real-time PCR

Real-time PCR was performed on 7900HT Fast Real-Time PCR System (Applied Biosystems, Foster City, USA) using Fast SYBR Green Master Mix (Applied Biosystems, Foster City, USA) according to the manufacturer's protocol.

### Co-immunoprecipitation

Nuclear protein from *NKX2-1*/*HOXB5* co-transfected SK-N-SH cells was prepared by NE-PER Nuclear and Cytoplasmic Extraction Reagent (Pierce Thermo Fisher, Rockford, USA), and pre-cleared with 50%-slurry of Protein G-Agarose. After pre-clearing, the slurry was incubated with mouse anti-HOXB5 antibody (133C3a, 25 µg; Santa Cruz, Santa Cruz, USA) or non-immunized mouse IgG control (5 µg; Santa Cruz, Santa Cruz, USA) at 4°C for 3 hours. The immune-precipitated protein complexes were resolved in a 8% SDS-Polyacrylamide gel before electro-transferred onto PVDF membrane. Mouse anti-NKX2-1 primary antibody (F-12, 1∶1,000; Santa Cruz, Santa Cruz, USA) and goat anti-mouse HRP-conjugated secondary antibody (Zymed, San Francisco, USA) were used in western blot detection. Signals were visualized by chemiluminescence using ECL Plus Western Blotting Detection Kit (GE Healthcare).

### Electrophoretic Mobility Shift Assay (EMSA)

Nuclear extract from *HOXB5* transfected SH-N-SH cells and HOXB5-GST fusion protein (Abnova, Taipei, Taiwan) were used in this study. End-to-end overlapping PCR fragments (generated using primers as shown in Table 1) spanning the D2 region of *RET* were labeled with Biotin-11-UTP (Pierce, Thermo Fisher, Rockford, USA). In brief, 25 fmole of Biotin-labeled DNA fragment was incubated with nuclear extract (7 µg) or HOXB5-GST protein (0.25 µg) with or without the addition of unlabeled fragment as specific competitor (300-fold in excess) in binding buffer (Lightshift Chemiluminescent EMSA Kit Pierce, Thermo Fisher, Rockford, USA) at room temperature for 25 minutes. Protein-DNA complex was resolved by electrophoresis on a 4.5% non-denaturing polyacrylamide gel, before being electro-transferred onto PVDF membrane, and signals were detected by chemiluminescence.

**Table 1:** Primers for PCR amplification.

Amplification	Primers	Annealing Temperature
Del.D (-1205 to 195)	*Forward: 5′- TCCTGCCTGTGAGGGTGAC-3′*	61°C
	*Reverse: 5′- AGTTGCTCTCCAGCGGTTC-3′*	
D1 (-1205 to -912)	*Forward: 5′-TCCTGCCTGTGAGGGTGAC-3′*	62°C
	*Reverse: 5′-GGGTGAGGTTGGATGTGGG-3′*	
D2 (-932 to -666)	*Forward: 5′-AGCCCACATCCAACCTCACC-3′*	62°C
	*Reverse: 5′-CCTCCAGCACTGGTCCAACC-3′*	
D3 (-700 to -363)	*Forward: 5′-TTTCCGGTTTCCAAGGGTTG-3′*	62°C
	*Reverse: 5′-CCTGCCTTTTGCCCTTTCC-3′*	
D4 (-387 to -115)	*Forward: 5′-TGCAGCGGAAAGGGCAAAAG-3′*	62°C
	*Reverse: 5′-CGCGTGTAGGAGCTCAGTG-3′*	
D2A (-932 to -666)	*Forward: 5′-AGCCCACATCCAACCTC-3′*	61°C
	*Reverse: 5′-CCTCCAGCACTGGTCC-3′*	

### Chromatin Immunoprecipitation

Chromatin immunoprecipitation (ChIP) was performed using MAGnify™ ChIP System (Invitrogen, Carlsbad, USA) according to the manufacturer's instruction. Briefly, 3×10^6^ *HOXB5/NKX2-1* transfected SK-N-SH cells were cross-linked in 1% formaldehyde and chromatin DNA was sheared by sonication (Vibra-cell VCX750 sonicator; 35% amplification, 20s ON plus 40s OFF, 25 cycles) to generate fragments of around 500 bp in length. Dynabeads® were pre-coupled with anti-HOXB5 (133c3a, Santa Cruz), anti-NKX2-1 (H-190, Santa Cruz), mouse or rabbit IgG. Sheared chromatin DNA was incubated with corresponding antibody-coupled beads at 4°C with rotation for 3 hours. After pull-down and reversion of cross-linking, chromatin DNA was purified. Binding of D2 to HOXB5 and NKX2-1 was quantified by real-time quantitative PCR analysis.

## Supporting Information

Figure S1 **A, Schematic diagram of the human** ***RET*** **promoter.** “+1” denoted transcription start of *RET* gene, and the first ATG of *RET* gene was indicated. B, Overlapping DNA fragments (D1 to D4) were generated by PCR and labeled with biotin for EMSA. Only D2 was able to bind to HOXB5 as shown by the retarded migration of the probe (arrow). C, Trans-activation of HOXB5 from D1 to D4 fragments were assayed by luciferase activity. Luciferase activity was normalized with Renilla luciferase to obtain relative luciferase unit. Fold increase (mean±SD) was determined relative to luciferase unit of empty vector (SV40-LUC) which was arbitrarily regarded as 1.
(TIF)

Figure S2 **No synergistic interaction between HOXB5 and PHOX2B, SOX10 or PAX3 in the trans-activation of** ***RET*** **promoter.** Wild-type full-length *RET* promoter was transfected with expression constructs of HOXB5, SOX10, PAX3 and PHOX2B either alone or in combination into SK-N-SH cells. Relative luciferase units for each combinations were determined, and fold increase (mean±SD) was calculated as compared to luciferase unit of empty vector (pXP1 Basic), which was arbitrarily regarded as 1.
(TIF)

Figure S3 **SK-N-SH cells express** ***RET*** **and** ***NKX2-1*** **, but not** ***SOX10*** **,** ***PAX3*** **and** ***HOXB5*** **.** Total RNA was isolated from SK-N-SH cells for RT-PCR analysis to assay for the expression of *RET*, *NKX2-1*, *SOX10*, *PAX3* and *HOXB5*. RT-PCR for *β-actin* was included as a positive control to test the integrity of the RNA and the RT-PCR reaction. PCR products were separated by agarose gel electrophoresis and visualized by ethidium bromide staining.
(TIF)
