# Supplementary figures and images for: HOXB5 Cooperates with NKX2-1 in the Transcription of Human RET
Source: PLoS One. 2011 Jun 3;6(6):e20815. doi: 10.1371/journal.pone.0020815 (PMC3108997; doi:10.1371/journal.pone.0020815)

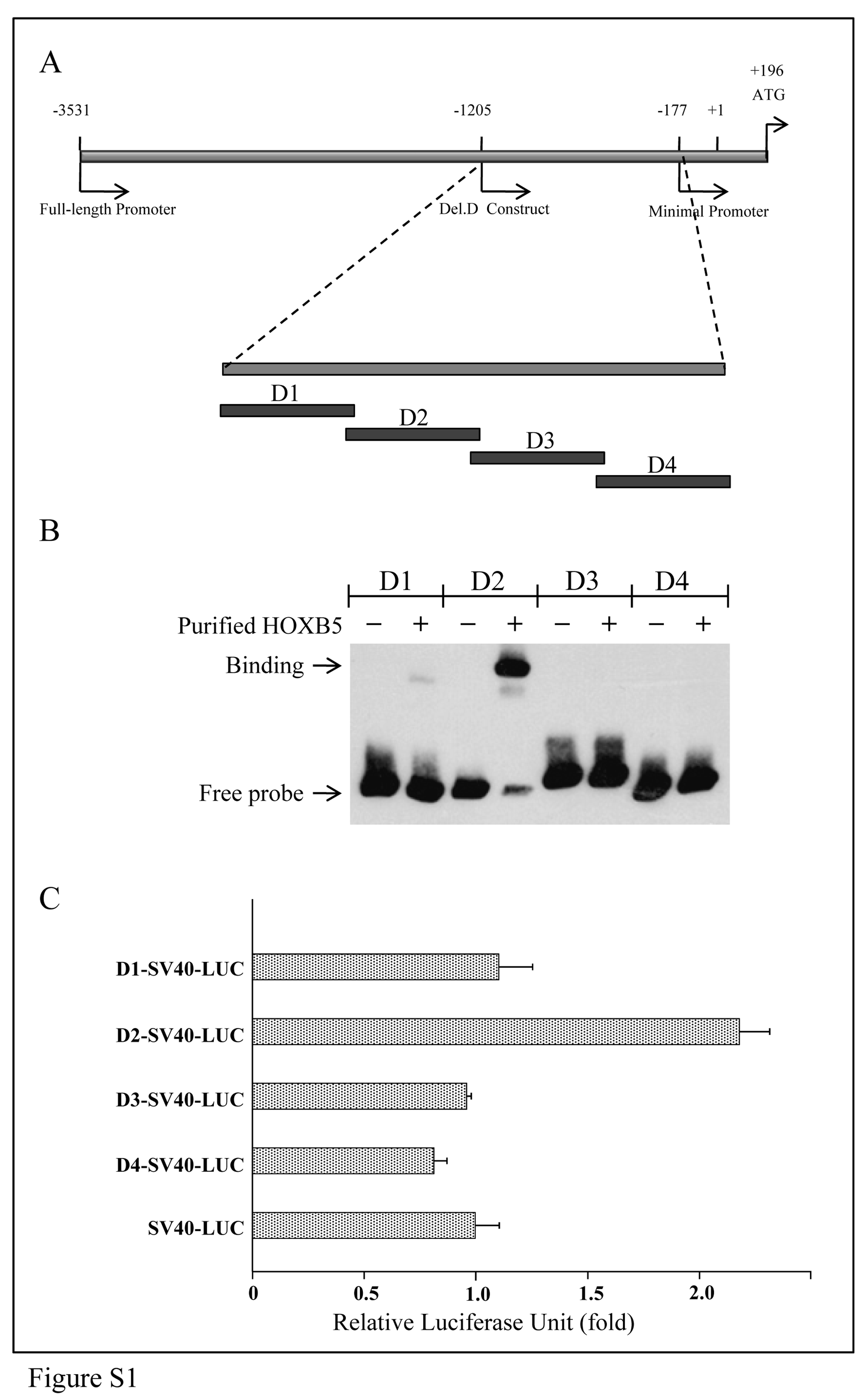

Supplement: Figure S1 — A, Schematic diagram of the human RET promoter. “+1” denoted transcription start of RET gene, and the first ATG of RET gene was indicated. B, Overlapping DNA fragments (D1 to D4) were generated by PCR and labeled with biotin for EMSA. Only D2 was able to bind to HOXB5 as shown by the retarded migration of the probe (arrow). C, Trans-activation of HOXB5 from D1 to D4 fragments were assayed by luciferase activity. Luciferase activity was normalized with Renilla luciferase to obtain relative luciferase unit. Fold increase (mean±SD) was determined relative to luciferase unit of empty vector (SV40-LUC) which was arbitrarily regarded as 1. (TIF) [file pone.0020815.s001.tif]

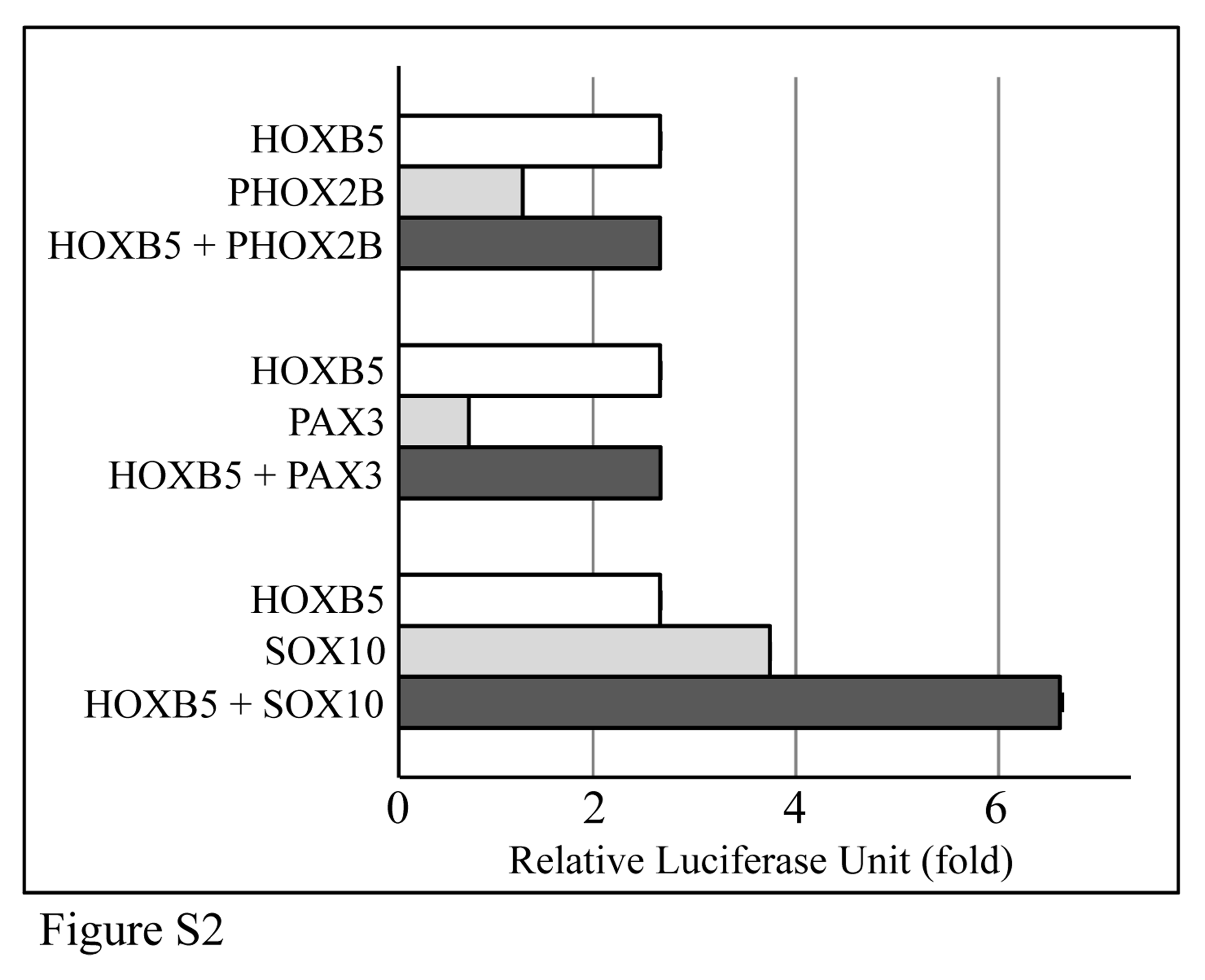

Supplement: Figure S2 — No synergistic interaction between HOXB5 and PHOX2B, SOX10 or PAX3 in the trans-activation of RET promoter. Wild-type full-length RET promoter was transfected with expression constructs of HOXB5, SOX10, PAX3 and PHOX2B either alone or in combination into SK-N-SH cells. Relative luciferase units for each combinations were determined, and fold increase (mean±SD) was calculated as compared to luciferase unit of empty vector (pXP1 Basic), which was arbitrarily regarded as 1. (TIF) [file pone.0020815.s002.tif]

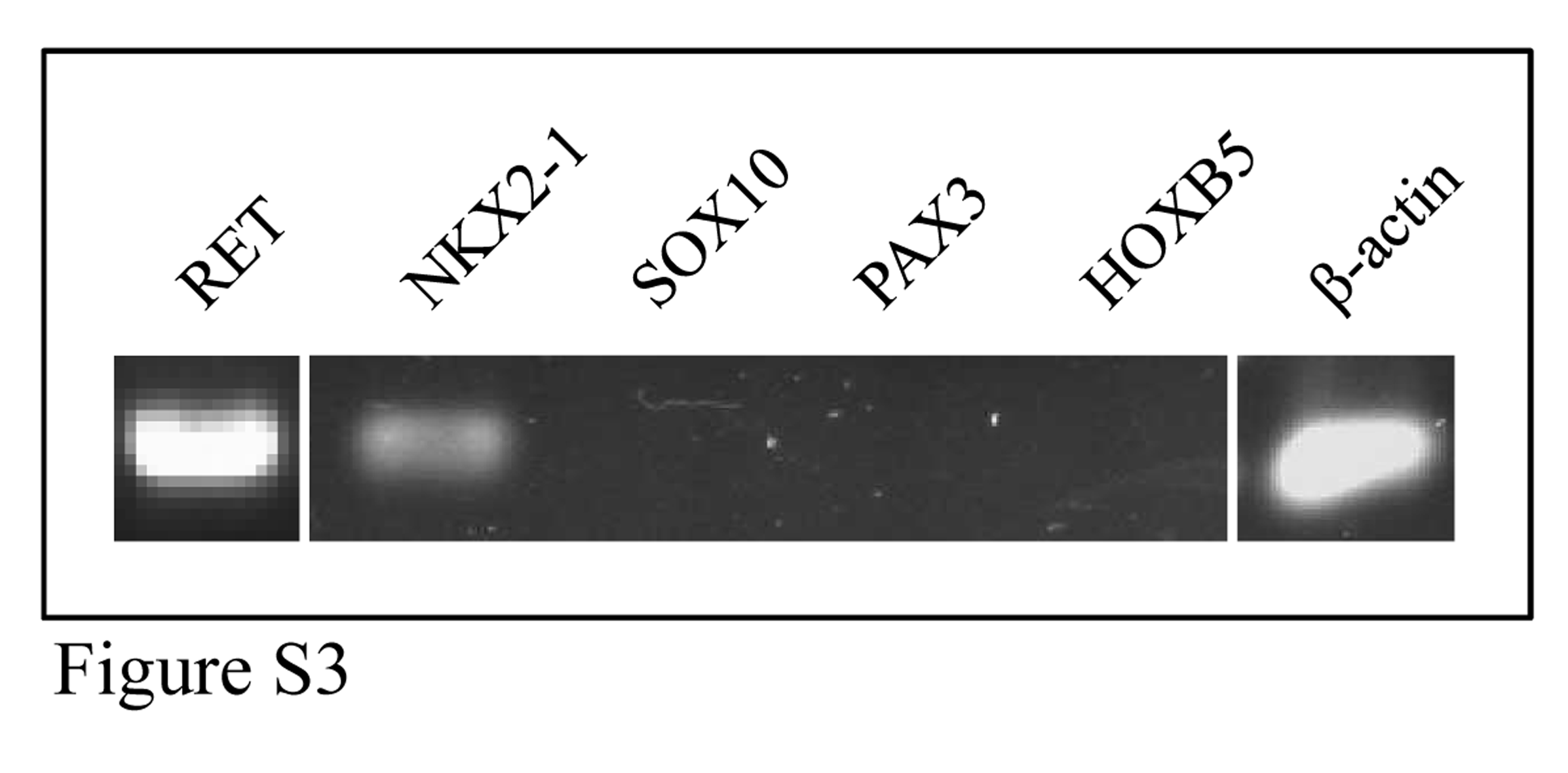

Supplement: Figure S3 — SK-N-SH cells express RET and NKX2-1 , but not SOX10 , PAX3 and HOXB5 . Total RNA was isolated from SK-N-SH cells for RT-PCR analysis to assay for the expression of RET, NKX2-1, SOX10, PAX3 and HOXB5. RT-PCR for β-actin was included as a positive control to test the integrity of the RNA and the RT-PCR reaction. PCR products were separated by agarose gel electrophoresis and visualized by ethidium bromide staining. (TIF) [file pone.0020815.s003.tif]
